# Mortality trends for young adults in Sweden in the years 2000–2017

**DOI:** 10.1177/14034948211000836

**Published:** 2021-03-25

**Authors:** Gunnar Ågren, Sven Bremberg

**Affiliations:** 1 Former National Institute of Public Health, Stockholm, Sweden; 2 Department of Global Public Health, Karolinska Institute, Stockholm, Sweden

**Keywords:** Mortality, young adult, Sweden, substance use, time trend

## Abstract

**Aim:**

Mental health problems in young people seem to be on the rise and more so in Sweden than in other locations. The aim was to compare the development of mortality rates for young adults in Sweden with Western Europe in total.

**Methods:**

Young adults were defined as individuals aged 20–34 years and the study period was 2000–2017. Mortality data were derived from the Institute of Health Metrics and Evaluation.

**Results:**

During the period 2000–2017, the mortality rate in young adults in Sweden stayed about the same, while in Western Europe as a whole the mortality rate decreased by 42%. The leading explanation for the unfavourable Swedish development was deaths due to drug use, mainly opioids, which increased by 60% during this period. The other major causes of death decreased both in Sweden and Western Europe, but decreased more slowly in Sweden. The differences in the rate of decrease between Sweden and Western Europe were for self-harm (27%), transport injuries (12%), unintentional injuries (31%) and for neoplasms (23%). The unfavourable development in Sweden resembled the development in the USA.

**Conclusions:**

**The risks of four of the five leading causes of death in this age group were affected by the individuals’ social conditions. The unfavourable mortality development in young adults in Sweden was mainly due to substance use. A contributing cause might be the change in the Swedish healthcare system that introduced competition between providers, which might have encouraged providers to prescribe opioids.**

## Introduction

The healthiest period of life, assessed as mortality rate, is adolescence and young adulthood. Yet, during recent decades in high-income countries, the decrease in mortality rates has been slower at 20–34 years of age compared with both younger and older age groups [1]. In this age group, about 20% of the burden of disease results from mental health disorders [1]. These problems seem to be on the rise in these countries [2]. The increase in mental health problems in young people has been especially apparent in Sweden [3] and in the Nordic countries as a whole [4]. Such an increase might be caused by increased strain in young people. However, it is not apparent why a rise in the level of strain should be more evident in Sweden than in other countries, since Sweden is a well-developed welfare society with social, educational and labour market spending well above the average for countries that belong to the Organisation for Economic Co-operation and Development (OECD), an organisation comprising mainly rich countries [5].

According to the World Values Survey, Swedes emphasise independence in their children more than people in most other countries [6]. Independence in young adulthood requires an income that will enable self-reliance. During the last decades, however, the period between childhood and the situation when a young person can support her- or himself by employment, has been extended [7]. This period, frequently referred to as emerging adulthood, is often problematic [8]. In Sweden in 1990, 75% of all males were able to sustain themselves at the age of 21, whereas in 2010 that was not the case until the age of 28 [9]. This extension seems to have evolved due to increased labour market demand for education, which has meant reduced demand for individuals with low qualifications [10]. Being unable to support themselves might be stressful for young adults, and more so in countries like Sweden that emphasise independence in young persons.

Increased strain in a population affects mortality rates. The dissolution of the Soviet Union, which began at the end of the 1980s, presents one such example. The collapse resulted in mass unemployment, a five-times increase in alcohol-related deaths and a doubling of suicide rates [11]. More recently, labour market developments in the USA have resulted in low educated individuals finding it increasingly difficult to secure employment. This has caused increased mortality rates, mainly due to alcohol and drug use disorders [12].

Thus, due to the apparent increase in mental health problems in Sweden [3], it was first hypothesised that the development of mortality rates in young adults in Sweden during recent decades would be more unfavourable than in comparable countries, that is, in Western Europe as a whole. Western Europe was chosen for comparison since the included countries are socially and economically similar to Sweden. Secondly, it was hypothesised that any unfavourable mortality development in Sweden would be due to deaths related to alcohol use, drug use and to suicide.

The aim of the study was to compare the development of mortality rates for young adults in Sweden and Western Europe.

## Methods

Young adults were defined as individuals aged 20–34 years and the period studied 2000–2017. All mortality data were derived from the Institute of Health Metrics and Evaluation (IHME) [1]. IHME includes data for the following 25 Western European countries: Andorra, Austria, Belgium, Cyprus, Denmark, Finland, France, Germany, Greece, Iceland, Ireland, Israel, Italy, Liechtenstein, Luxembourg, Malta, Monaco, the Netherlands, Norway, Portugal, San Marino, Spain, Sweden, Switzerland and the UK. In addition, some comparisons were also done with the US since an unfavourable mortality development in this country has recently been highlighted [12].

In the IHME system, the diagnosis groups are based on the codes in the World Health Organization’s International Classification of Diseases (ICD) system. During the study period, ICD-10 was mainly used. However, there were some differences between the IHME diagnosis groups and ICD-10. For example, IHME’s ‘self-harm’ excludes intentional self-poisoning by and exposure to alcohol (ICD-10 X65) and includes assault by pesticides (X87). IHME’s ‘substance use disorder’ excludes use of tobacco (F17) but includes degeneration of nervous system due to alcohol (G31.2); findings of drugs and other substances, not normally found in blood (R78); accidental poisoning by and exposure to alcohol (X45); intentional self-poisoning by and exposure to alcohol (X65); and poisoning by and exposure to alcohol, undetermined intent (Y15). For IHME ‘transport injuries’, ‘unintentional injuries’ and ‘neoplasms’ there are also some relatively minor differences between the IHME and ICD-10 classification systems. Details about the differences between IHME and ICD-10 and ICD-9 classification systems are available on the IHME website [1]. The differences between the ICD classification system and that used by IHME were not expected to have a substantial effect on the analysis that was performed.

The IHME system retrieves data from official national mortality databases that are listed on the IHME website [1]. A computer algorithm smooths out differences between years. No age standardisation was employed. Since this study was completed, IMHE has published an update that now includes the years 2018 and 2019. Data from this update were not included in the current study.

No statistical analysis was performed.

## Results

**Figure 1.**
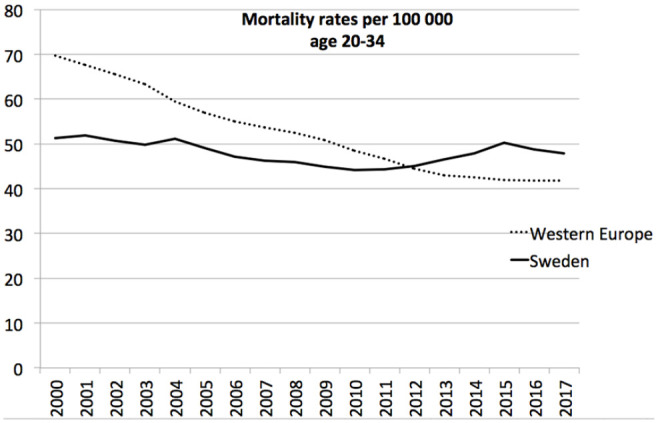
Mortality rates at the age of 20–34 in Sweden and Western Europe in the period 2000–2017.

The developments in mortality rates at the age of 20–34 in Sweden and Western Europe in the period 2000–2017 are presented in Figure 1. In Western Europe, mortality rates fell steadily to 42% while in Sweden the rates stayed about the same. Since IHME employs an algorithm to smooth out differences between individual years, peaks are less apparent. Figure 1, nonetheless, indicates a slight mortality peak in Sweden around 2015.

**Figure 2.**
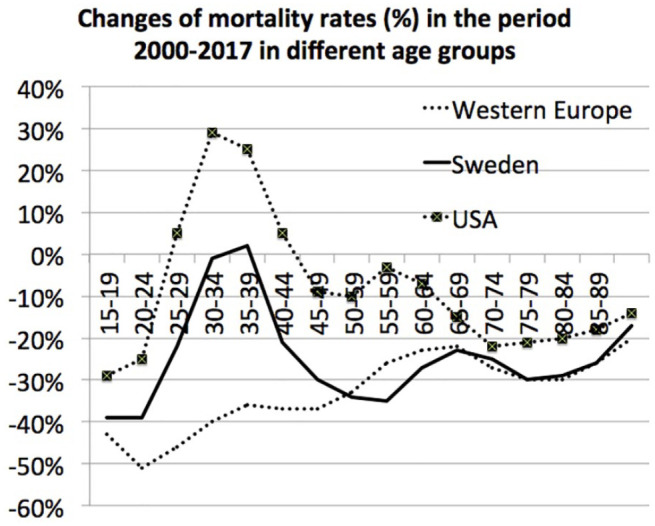
Mortality rates in the year 2017 compared with mortality rates in the year 2000, in three different age groups, in Sweden, Western Europe and the USA. Changes are presented as the rate in 2017 as a percentage of the rate in 2000.

The changes in mortality rates in 2000–2017 in different age groups in Sweden and Western Europe are presented in Figure 2. In the figure, the corresponding changes in the USA are also included. In Western Europe, the mortality rates decreased in all age groups but most in the youngest age groups. In Sweden for most ages, the mortality rates also decreased. Yet the 20–39 age group was an exception with much slower rates of decrease. In the USA, the mortality rates also decreased but more slowly than in both Sweden and Western Europe. The 20–39 age group in the USA was also an exception with increasing mortality rates. Thus, the mortality peak not only comprised individuals aged 20–34 but also 35–39 year-olds. The study, however, focused on 20–34 years of age since these ages are included in the concept ‘emerging adulthood’ [7], as described in the Introduction.

Table I presents the percentage change in mortality rates in Sweden and Western Europe at the age of 20–34, in the period 2000–2017 and for the five most common causes of death in this age group. Deaths due to substance use disorders increased in Sweden. All other common causes of death decreased both in Sweden and Western Europe. The rates of decrease for the remaining four causes were, however, lower in Sweden than in Western Europe.

**Table I.** Mortality rates per 100,000 at the age of 20–34 in 2000 and 2017 in Sweden and Western Europe.

Cause of death	Sweden			Western Europe			(*a*)*–*(*b*) (%)
	2000	2017	*Change* (*a*) (%)	2000	2017	*Change* (*b*) (%)	
Self-harm	14.45	14.33	−0.01	12.62	9.17	−27.3	27.2
Substance use disorder	5.17	8.26	+59.6	5.31	4.28	−19.4	79.0
Transport injuries	9.36	4.68	−50.0	18.9	7.21	−61.9	11.9
Unintentional injuries	3.46	3.06	−11.6	5.03	2.89	−42.5	30.9
Neoplasms	7.35	7.33	−0.02	9.58	7.33	−23.3	23.1
All causes	51.37	47.87	−6.80	69.68	41.82	−40.0	33.2

**Figure 3.**
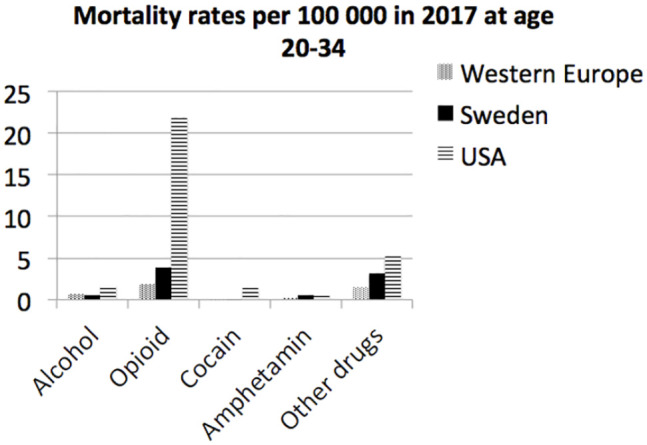
Mortality rates per 100,000 in 2017 due to different substance use disorders at the age of 20–34 in Western Europe, Sweden and the US.

Since substance use disorders were the dominant cause of the increased deaths at the age of 20–34 in Sweden, the rates for different substances in 2017 are presented in Figure 3 together with data from Western Europe and the USA. Deaths due to opioid use disorders dominated in Sweden as well as in the USA and Western Europe. Deaths due to cocaine were almost neglectable in Western Europe and Sweden and deaths due to amphetamine were negligible in Western Europe.

## Discussion

During the period 2000–2017, the mortality rates in young adults (aged 20–34 years) developed more unfavourably in Sweden compared with Western Europe. A potential explanation for this pattern might be that both Sweden and Western Europe had reached an optimal level at the end of this period. However, the mortality rates in Sweden did not only stagnate; they also reached levels above Western Europe during the last 5 years of the study.

The leading explanation for the unfavourable Swedish development was deaths due to drug use. All other major causes of death also developed more unfavourably in Sweden, although to lesser extent. The risks for all four of the five leading causes of death were affected by the individuals’ social conditions. That is true of deaths due to drug use [13], unintentional injuries [14], transport injuries [15], and self-harm [16]. These causes of death support the notion that young Swedes might be exposed to certain kinds of strain that have been less apparent in Western Europe. Deaths due to alcohol use disorders are also affected by social conditions [17]. This problem, however, contributed relatively little to the causes of death in 2017 in young adults in Sweden (0.6 deaths per 100,000).

### Sweden and the US

A similar development of mortality rates in young adults in the US is demonstrated in Figure 2. This development in the USA has previously been reported [12]. Thus, in Sweden and in the USA, similar age groups are affected. Both in Sweden and in the USA, the dominant cause of death has been drug use disorders. This increase in drug deaths has been related to a deteriorating labour market for low educated persons [10,12].

The resemblance between Sweden and the USA was unexpected since the welfare systems in Sweden are much more developed than in the USA. This difference is reflected in social spending in 2018 as a proportion of gross domestic product that in Sweden amounted to 26.1% and in the USA to only 18.7% [5]. Yet, the welfare systems in Sweden are mainly aimed at children, working-age adults and old-aged people. Young adults, who often have not entered regular employment, have not been prioritised, for example, by means of specific educational or financial forms of support. That is reflected in the proportion of the population in Sweden with incomes that are low enough to qualify for welfare. In 2012, at 16–24 years, 38% qualified; at the age of 25–34 it was 11%. These figures might be compared with the fraction that qualified for welfare at the age of 65–74, 3%, and at 75–84 years of age, 4% [18].

Thus, the similarity between Sweden and the USA might be due to the welfare system failing to adapt to a situation where most persons do not secure employment that have incomes that enable them to sustain themselves until about the age of 30. However, this cannot be the only explanation since a negative mortality development in young adults was not seen in other parts of Western Europe.

### The educational system

During the last decades, level of education has gained increasing importance in relation to mortality rates in Europe, especially in the Nordic countries [19]. The educational mortality gradient affects young adults more than other age groups. In an Estonian study, the educational mortality gradient during the period 1989–2000 increased more in the 20–39 age group compared with all other age groups [20]. Similarly, in an English area-based study, the social inequalities in mortality were relatively low during adolescence, started to increase at the age of 20, reached a peak around the age of 30, and thereafter gradually diminished to zero at 80 years of age [21].

In Sweden, educational achievement at the end of compulsory schooling has, since the end of the 1990s, deteriorated faster than in any other OECD country [5]. This development might have contributed to an unfavourable mortality development in young Swedes during a period when the importance of educational achievement has increased.

A peculiarity in the Swedish educational system was introduced in 1990 [22]. At that time, vocational schools and theoretical secondary schools were merged into a single system. All education at that level had to prepare individuals for tertiary education. That meant that students with failing grades in primary school were not admitted. As vocational training is not offered outside the secondary school system, substantial groups of low achieving students are not offered any vocational training. In 2019 this group encompassed 16% of all students [23]. Sweden is probably the only country in Western Europe that excludes such a large group from vocational training. This aspect of the Swedish educational system might have increased the vulnerability of the most disadvantaged young people.

### Employment

The negative mortality development in young adults in the USA has been ascribed to increasing demands for education [12]. This development is true of most high-income countries [10]. Yet, according to OECD, the job requirements in the USA, Sweden and Northern Europe are quite comparable and so are both employment and unemployment rates at the age of 25–29 [5]. Thus, there is no variation in skills requirements for employment, or in employment rates, that might explain why mortality increased in the USA, levelled out in Sweden and decreased in Western Europe.

### Drug deaths

The leading cause of death at the age of 20–34, in Sweden, Western Europe and the USA was substance use disorders. The mortality rates were highest in the USA followed by Sweden and Western Europe.

Mortality due to drug use disorders might be related to drug prescriptions. Prescription rates in Sweden in the period 2000–2017, assessed as daily doses per 1000 inhabitants, were compared with the rates in the OECD countries as a whole [5]. The rates in Sweden for nervous system drugs in total (ATC code N) were 1.53 times the OECD rate; for analgesics (N02) 2.34 times the OECD rate; for anxiolytics (N05b) 0.65 times the OECD rate; for hypnotics and sedatives (N05C) 1.92 times the OECD rate; and for antidepressants (N06A) 1.48 times the OECD rate. The prescription of opioids (N02A) in Sweden to patients aged 20–34 during the period 2006–2017 increased from 113.6 to 127.6 per 1000 inhabitants, that is, by 12% [24]. However, according to the more detailed Swedish pharmaceutical registry, the number of prescriptions of the opioids responsible for most drug-related deaths (Morphine N02AA1, Oxycodone N02AA05, Fentanyl N02AB01, Buprenorphine N02AE01, N07BC01 and N07BC51, Methadone N07BC02) increased from 17.9 to 61.3 per 1000 inhabitants aged 20–34 during this period [24].

The prescription of drugs might be affected by the design of the healthcare system. A national law to enable citizens’ freedom of choice of care provider was passed in 2008 that included healthcare [25]. One of the objectives of this act was to expand the provision of private healthcare, and another to increase competition among providers, both public and private [26]. This reform might have contributed to increased drug prescription as all providers now have to compete for patients.

The rate of deaths that is ascribed to drug use might be affected by autopsy practices. In Sweden, forensic investigations, including toxicological analyses, are routinely carried out when individuals <65 years of age die of unnatural causes; autopsy practice is less meticulous in many other Western European countries [27]. This difference might explain part of the higher rate of drug deaths in Sweden compared with Western Europe but cannot explain the higher overall mortality rate.

Substance use deaths in Sweden will be analysed in separate a paper. Drug policies vary within Western Europe. In some restrictive countries like Sweden, possession of illegal drugs is always prosecuted, while in other nations like the Netherlands, possession of small amounts of certain drugs is not considered to be a criminal offence [28]. The criminalisation of drug possession is expected to reduce drug use [29]. Criminalisation, however, might also reduce help-seeking [30]. The policies that regulate the distribution of antidotes also vary. Since there is no consensus on the overall effect of drug policies, it is hard to decide whether the restrictive Swedish drug policy has contributed to the unfavourable mortality development in Sweden.

### Strengths and weaknesses

The analysis builds on official death registers. The total figures in high-income countries are very reliable. Thus, there is little doubt about the validity of the main finding of increased mortality in young adults in Sweden in contrast to Western Europe as a whole.

The main weakness is the lack of explanations for the young adult mortality increase. Further research is required.

## Conclusions

In Sweden, the mortality rates in young adults have increased during the last decades. This development is unique for Sweden in Western Europe but similar to the development in the USA.
